# Stillbirth Risk during the 1918 Influenza Pandemic in Arizona, USA

**DOI:** 10.3390/epidemiologia1010005

**Published:** 2020-11-11

**Authors:** Smriti Khare, Sushma Dahal, Ruiyan Luo, Richard Rothenberg, Kenji Mizumoto, Gerardo Chowell

**Affiliations:** 1 Department of Population Health Sciences, School of Public Health, Georgia State University, Atlanta, GA 30303, USA; skhare2@student.gsu.edu (S.K.); rluo@gsu.edu (R.L.); rrothenberg@gsu.edu (R.R.); gchowell@gsu.edu (G.C.); 2 Graduate School of Advanced Integrated Studies in Human Survivability, Kyoto University Yoshida-Nakaadachi-cho, Sakyo-ku, Kyoto 606-8306, Japan; mizumotokenji@gmail.com; 3 Hakubi Center for Advanced Research, Kyoto University, Yoshidahonmachi, Sakyo-ku, Kyoto 606-8306, Japan

**Keywords:** influenza pandemic, stillbirth risk, mortality, 1918–1919 influenza, Arizona, maternal age

## Abstract

The 1918 influenza pandemic, the deadliest pandemic on record, affected approximately 1/3rd of the population worldwide. The impact of this pandemic on stillbirth risk has not been studied in depth. In this study, we assessed the stillbirth risk during the 1918 influenza pandemic in Arizona, USA. We carried out a retrospective study using 21,334 birth records for Maricopa County, Arizona, for the period 1915–1925. We conducted logistic regression analyses to assess the effect of that pandemic on stillbirth risk. Though we did not find a statistically significant impact on stillbirth risk during the pandemic, there was a higher risk of stillbirth in July 1919 (42 stillbirths/1000 births), 9 months after the peak pandemic mortality, and a stillbirth risk of 1.42 (95% CI: 1.17, 1.72) in women ≥35 years compared to the women aged <35 years. The risk of stillbirth was lowest if the mother’s age was approximately 26 years at the time of birth. We also report peaks in stillbirth risk 9–10 months after the peak pandemic mortality. Our findings add to our current understanding of the link between pandemic influenza and stillbirth risk.

## 1. Introduction

The 1918 influenza pandemic, also known as Spanish flu, is considered to be the deadliest known pandemic and caused an estimated 50–100 million deaths globally [1]. Compared to other influenza pandemics, this pandemic had many unusual features. It was exceptionally severe, with a high case fatality rate estimated at 2.5% [1]. The highest mortality rate during this pandemic was among young healthy adults aged 20–40 years [1,2,3] and it unfolded in a sequence of waves between 1918 and 1920 [4].

Several studies have documented the severe effects of influenza among pregnant women [5,6] and increased risk of fetal death [7]. The severe outcomes of influenza infection during pregnancy include low birthweight [8], preterm birth, pregnancy termination [9,10], and death of pregnant women [11]. Lieberman et al., reported a causal relationship between early exposure to influenza infection during pregnancy and fetal death based on evidence of transplacental transmission of virus from mother to fetus [10]. Studies have also documented a high mortality and decline in birth rates during the 1918 influenza pandemic [12,13]. A study set in Arizona, USA, found a significant decline in birth rates 9–11 months after peak pandemic mortality during the 1918 influenza pandemic [14]. However, the impact of the 1918 influenza infection on the risk of stillbirth remains poorly studied. The CDC defines stillbirth as the death or loss of a baby after 20 weeks of pregnancy that can occur before or during the delivery. Based on when it occurs, it can be classified as an early, late, or term stillbirth [15]. The understanding of the risk of stillbirth is important, since influenza vaccination is currently promoted during pregnancy [14]. Vaccination during pregnancy decreases the risk of influenza infection in women [7] and reduces the hospitalization of their babies (aged <6 months) for influenza [16]. Addressing the elevated risk of complications associated with the influenza infection, WHO currently recommends that pregnant women should be given the highest priority for seasonal flu vaccinations [17].

A study reported that the 1918 influenza pandemic killed 0.8% of Arizona’s population [18], which falls in the upper range of estimates previously reported for several U.S. settings [19,20,21]. In this paper, we used information of 21,334 individual birth certificates which were carefully retrieved from the publicly available Arizona genealogy database to assess the impact of the 1918 influenza pandemic on the risk of stillbirth in Maricopa county, the most populous county of Arizona. In addition, based on earlier work [20] suggesting an increase in risk of stillbirth with the increase in maternal age, we also aim to assess the risk of stillbirth with advancing maternal age.

## 2. Materials and Methods

Data source: Arizona Genealogy Database (http://genealogy.az.gov/) is generated by the Arizona Department of Health Services. In this database, birth records for the years 1855 to 1943 are publicly available online. For this study, we manually retrieved 21,334 birth records from January 1915 to December 1925 for Maricopa County, Arizona. For each birth record, we extracted information on birth date, birth status of the child, and age of the mother. Birth status was recorded as either alive, stillbirth, or missing.

Statistical analysis: We estimated the stillbirth risk as total number of stillbirths per 1000 births (live birth plus stillbirth). To define the pandemic period in Maricopa, Arizona during 1918 influenza pandemic, we referred to a study that evaluated the mortality patterns in Arizona during the pandemic [18]. The study used the time-series of Pneumonia and Influenza (P&I) mortality and reported that Arizona experienced three successive waves of increased mortality from 1918–1920. The first herald wave occurred in April of 1918; the second, prolonged wave from fall 1918 (October–December 1918) to winter 1919 (January–April 1919); and a third wave in in the winter of 1920 (February–April 1920) [18].

We also categorized the study period from 1915 to 1925 as ‘Pre-Pandemic’ (January 1915 to March 1918), ‘Pandemic’ (April 1918 to April 1920 + 9 months) and ‘Post-Pandemic’ (February 1921 to December 1925). We added 9 months to the ‘pandemic’ period to account for the duration of pregnancy considering the delayed impact of influenza on birth outcomes.

We estimated the risk of stillbirths per 1000 births for the pandemic period. We plotted it for each month from April 1918 to January 1921 to inspect the temporal pattern during the three consecutive pandemic waves. To observe the effect of the pandemic on stillbirth risk and to assess the association between maternal age and stillbirth risk, we used logistic regression (SAS, version 9.4. SAS Institute Inc., Cary, NC, USA). We assessed the association using two separate models with birth status (Alive = 1 and Stillbirth = 0) as the outcome. In the first model, we evaluated the impact of the pandemic by categorizing the study period as ‘pre-pandemic’, ‘pandemic’, and ‘post-pandemic’. In the second model, we built a linear spline regression model with three linear splines of time with knots on 31 March 1918 and 31 January 1921, which allowed us to check whether there were changes in slopes from the pre-pandemic to pandemic, and from the pandemic to post-pandemic period. Both models included maternal age and quadratic term of maternal age as covariates.

We considered age in three ways: (a) categorical with two groups: <35 and ≥35, (b) categorical with four groups: 10–19 years, 20–29 years, 30–39 years, 40 years and above, and (c) continuous. The binary category was selected based on a previous systematic review in which the most commonly used definition of advanced maternal age was found to be 35 years or more for older women and less than 35 years for younger women [22]. We then evaluated the risk of stillbirth for women in these two categories. We also ran logistic regression analysis to evaluate the association of risk of stillbirth and maternal age (continuous variable). Since a non-linear relationship was observed, both linear and quadratic terms of maternal age were included in the model

Logit(P(Stillbirth)) =  b0+b1 Maternal age +  b2(Maternal age)2

where −$b_{1}$/(2$b_{2}$) gives the maternal age at which the risk of stillbirth is the least when $b_{2}$ > 0.

Since we had birth records with missing birth status in our dataset, we also conducted a sensitivity analysis, where we imputed the records with missing birth status with the stillbirth indicator. This sensitivity analysis did not yield significantly different results.

## 3. Results

We analyzed 20,838 of the 21,334 birth records retrieved for the study period after excluding 2.3% of the birth records with missing birth status or missing maternal age. Of the total missing records, 114 (22.98%) were due to missing birth status while 388 (78.22%) were due to missing maternal age. Of the total 114 records with missing birth status, 16.67% were in pre-pandemic period, 34.21% in pandemic period, and 49.12% in post-pandemic period.

We found that the year 1920 had the highest proportion of births (12.6%). The years 1922 and 1917 experienced the highest (3.3%) and the lowest proportion (2.4%) of stillbirths, respectively, in the study period. In the entire study period from 1915 to 1925, we saw a slight increase in the risk of stillbirths during the pandemic period (1918–1920).

We found that the risk of stillbirth was significantly higher among older women (≥35 years) (Relative Risk: 1.42, 95% CI: 1.17, 1.72) compared to the younger women (<35 years) (Table 1).

Table 2 shows that during the study period, 54.3% births occurred among the mothers of age group of 20–29 years, followed by 28.9% births among the age group of 30–39 years. The results show that the relationship between stillbirth risk and maternal age is not linear. The mothers of age 40 years and above had the highest percentage of stillbirths (3.5%) followed by mothers of the age group 30–39 years (3.1%). The percentage of stillbirths was the lowest for the mothers of the age group 20–29 years (2.6%).

We found a significant association between stillbirth risk and quadratic term of maternal age at 0.05 level of significance with *p*-value of 0.009 (Table 3). The risk of stillbirth was lowest when the mother’s age was 26 years at the time of birth.

Table 4 shows the findings from multivariate analysis for the association of stillbirth risk and time. The left part of the table includes time as a categorized variable, whereas the right part of the table includes time as a continuous variable with linear spline effects. The results of logistic regression did not show a significant association of the pandemic period with the risk of stillbirth compared to the pre-pandemic and post-pandemic period, but the correlation with maternal age remained significant. Additionally, the change in slope for the stillbirth risk from pre-pandemic period to pandemic period was not significantly different from zero, but the relationship with the mother’s age remained significant (Table 4).

**Figure 1:**
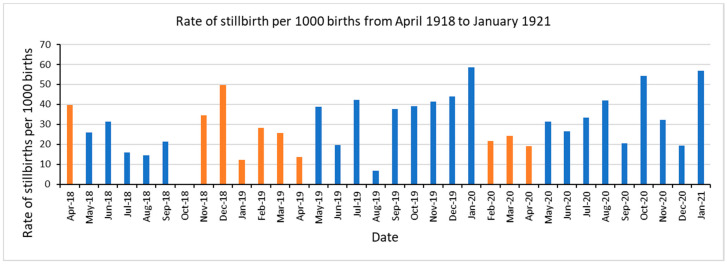
Monthly rates of stillbirth per 1000 births in Maricopa, Arizona from April 1918 to January 1921. Orange bars reflect the time of three successive pandemic waves in Arizona April 1918, October 1918–April 1919, and February 1920 to April 1920 [18].

Figure 1 represents the rate of stillbirths per 1000 births for the pandemic period from April 1918 to January 1921. The orange bars reflect the time of pandemic waves: the first wave in April 1918, second long wave from October 1918 to April 1919, and third wave from February 1920 to April 1920 [18]. We observed a higher rate of stillbirths of ≥50 per 1000 births in December 1918 (49.7~50), January 1920 (58.5), October 1920 (54.4), and January 1921 (56.8). There were no stillbirths recorded in October 1918.

## 4. Discussion

In this study, we assessed the impact of the 1918 influenza pandemic on stillbirth risk in Maricopa county, Arizona. We also assessed the association of stillbirth risk with the age of the mother. We expected an increased rate of stillbirths during the pandemic, due to the biological and physiological effects of the pandemic infection on pregnancy. We referred to the previous work by Dahal et al. to define the pandemic waves in Arizona [18].

We did not find a statistically significant effect of the 1918 influenza pandemic on the stillbirth risk. One previous study reported a statistically significant association of the pandemic with stillbirth outcome in Japan [23]. Previous studies have reported peaks in stillbirth risk as well as the risk of spontaneous abortions that coincide with peaks in pandemic mortality [13,24,25]. Interestingly, October 1918, the month of peak pandemic mortality, did not record any cases of stillbirth. This could be attributed to underreporting of stillbirths, especially during peaks in the pandemic. During the pandemic period (April 1918 to April 1920), most of the months in the second and third pandemic wave reported lower stillbirth rates than that of the months without the pandemic wave.

We suspected that the majority of missing birth status information could be due to stillbirths. Therefore, we repeated the analysis after accounting for all the missing birth status records as stillbirths. However, we did not find significant differences in our results and there was not a statistically significant effect of the 1918 influenza pandemic on the stillbirth risk.

We report an intriguing pattern in the stillbirth rate during the pandemic period. Specifically, the observed peaks in stillbirth rates in December 1918 occurred at 9 months after the first herald wave in April 1918. Similarly, the observed peak in July 1919 was at 9 months after November 1918, the mid of the fall 1918 wave. The peak in stillbirth in December 1919 and January 1920 was 9–10 months after the winter 1919 wave. Likewise, the observed peak in October 1920 was at 9 months after the start of winter 1920 wave. This finding is consistent with the natality decline reported in a prior study conducted in Arizona using the same data source [14], in which a decline in birth rate by 43% was observed in July 1919, 9–10 months after the peak pandemic mortality during the fall wave. The simultaneous decline in birth rates and an increase in stillbirths at 9–10 months of pandemic waves, could be attributed to influenza infection in mothers during the first trimester of their pregnancies. In Norway, the increase in stillbirths has been explained by Mamelund S-E to be due to: (a) a larger proportion of conceptions than normal were out of wedlock at the end of 1918 and 1919, and illegitimate births could be associated with a higher risk of a stillbirth; (b) the 1918 influenza also selected the young (peak around 30 years) mothers in 1918, leaving a greater proportion of the births in 1919 to older women who exhibit a higher stillbirth risk, and (c) with a higher sex ratio at birth with more boys than girls being conceived after war and influenza, it might be expected to see a rise in stillbirth rates, because men have higher mortality than women at all ages [26]. We note that our results could also be explained by these mechanisms.

In our study, we did not find any seasonality in our data on stillbirth rate for the period from April 1918 to December 1920. We found a significant association between the age of the mother and the risk of stillbirths, and this is in line with the literature, where there is an increased risk of stillbirths for women with advanced maternal age [22,27]. A systematic review by Huang et al. found that in 24 of 31 cohort studies and all six of case-control studies, the risk of stillbirth increased with an increase in maternal age, with a relative risk that varied from 1.2 to 4.3 in older versus younger women [22]. In our study, we found a stillbirth risk of 1.42 in older women compared to women less than 35 years of age. Another study by Reddy et al. [27] documented a higher risk of stillbirth with advanced maternal age; the relative risk of stillbirth was 1.32 and 1.88 for women 35–39 years and women older than 40 years of age, respectively, when compared to women less than 35 years of age. Additionally, we observed a significant association between stillbirth risk and maternal age (*p*-value < 0.050) and found that the risk of stillbirth is least when the mother’s age at birth is approximately 26 years.

This study is retrospective in design, and the birth records are approximately a hundred years old. We lack data or information on the health status of the mothers, and if the mothers were clinically infected during pregnancy or not. Since Arizona experienced high mortality rates at the time, the study assumes that a high proportion of pregnant women were infected during the pandemic. Another limitation to the study is the misclassification of birth status on the birth records; since these birth records are images of handwritten birth forms, many records were left blank for alive/stillbirth status by the health care professional in charge. We classified them as alive if the child was named or had an additional record/certificate uploaded for the change in the name of the child. This could lead to misclassification and could have underestimated the results. Our finding of no stillbirth record in October 1918, the time of peak pandemic mortality, could be an artifact of reporting-recording. Additionally, Arizona did not participate in the US vital registration until 1926; the dataset retrieved from genealogy database cannot be compared with the official statistics for the study period for Arizona.

The study employed statistical analyses and a large sample size to assess the impact of pandemic on stillbirth outcomes. However, we did not include other factors that could have had an impact on stillbirth risk. For example, Maricopa County experienced higher rates of tuberculosis than the rest of the United States [28] until the 1950s due to the continued migration of people suffering from tuberculosis, because of its dry climate, believed to be favorable for its treatment [28,29]. We did not include other factors such as pre-existing respiratory illness/tuberculosis that could have potentially led to high stillbirth rates throughout the study period. Our study did not include the effects of other important factors like parity, mother’s work during pregnancy, previous history of stillbirths, and other demographic and socioeconomic factors, as found by Reid A [30]. According to a meta-analysis, the risk of stillbirth in subsequent pregnancy is higher for women with a history of stillbirth [31]. Miscarriages and stillbirths during the pandemics also affect the subsequent fertility rates of the affected regions. For example, the women who undergo conditions such as miscarriages and stillbirth are temporarily infecund, thereby reducing the overall natality of the affected regions for a certain period after the pandemic waves [25,32]. However, we have not explored the potential confounding effect of changes in birth outcomes due to stillbirths. Future studies on influenza pandemic should consider including these variables to better understand the association between influenza infection among pregnant women and risk of stillbirths.

Studies have reported that influenza infection adversely affects the pregnancy outcomes including abortion and stillbirth especially if the infection occurs in the first trimester [6,9,11]. Our findings also showed that there was a surge in stillbirth cases 9–10 months after the peak in pandemic in the fall 1918 wave, as well as at 9–10 months after the first herald wave, after the winter 1919 wave, and after the start of winter 1920 wave. Although we could not ascertain whether that surge in number of cases was due to influenza infection, we support the influenza vaccination recommendation during pregnancy.

## 5. Conclusions

Assessment of stillbirth risk during an influenza pandemic is an important indicator of the direct effect of the influenza virus on the fetus. Knowledge of the risk of stillbirth also has important applications in terms of policies related to the vaccination of pregnant women. From our detailed analysis of birth records from January 1915 to December 1925 for Maricopa county, Arizona, we did not find a statistically significant impact of the 1918 influenza pandemic on stillbirth risk. However, we found peaks in stillbirth rate 9–10 months following the pandemic waves. Additionally, our results are in agreement with the literature, documenting a greater stillbirth risk associated with older maternal age. This is the first study conducted in Arizona to assess the risk of stillbirths associated with the deadliest pandemic to date. We recommend further studies to conclude the effect of influenza infection on fetus and the risk of stillbirths.

## Figures and Tables

**Table 1:** Maternal age category by birth status.

Maternal Age Category	Birth Status			Relative Risk (95% Confidence Interval)
	Stillborn	Alive	Total	
Old (≥35 years)	123	3105	3228	1.42 (1.17, 1.72)
Young (<35 years) (reference group)	473	17,137	17,610	1.00
Total	596	20,242	20,838	

**Table 2:** Birth status by maternal age group.

Birth Status		Maternal Age Group				
		10–19 Years	20–29 Years	30–39 Years	40 Years and above	Total
**Alive**	Frequency	2505	11,013	5846	878	20,242
	Percentage	12.0	52.8	28.0	4.2	97.1
	Column %	97.0	97.3	96.9	96.5	
**Stillborn**	Frequency	77	300	187	32	596
	Percentage	0.4	1.4	0.9	0.1	2.9
	Column %	2.9	2.6	3.1	3.5	
**Total**	Frequency	2582	11,313	6033	910	20,838
	Percentage	12.4	54.3	28.9	4.4	100.0

**Table 3:** Logistic regression analysis for the association of stillbirth risk and maternal age.

Variable	Beta Estimate	Standard Error	*p*-Value
Maternal age	−0.10	0.05	0.02 *
(Maternal age)^2^	0.002	0.00	0.009 *

* Significant at 0.05 level of significance.

**Table 4:** Multivariate analysis for the association of stillbirth risk and time.

Time as a Categorical Variable	Beta Estimate	*p*-Value	Adjusted OR (95% CI)	Time as a Continuous Variable	Beta Estimate	*p*-Value
Pre-pandemic period	1.00 (ref)			Time *	−0.04	0.59
Pandemic period	0.06	0.63	1.06 (0.84, 1.35)	Z1 **	0.11	0.36
Post-pandemic	−0.07	0.53	0.93 (0.75, 1.16)	Z2 ***	−0.13	0.10
Maternal age	−0.10	0.02 **		Maternal age	−0.10	0.02 ****
(Maternal age)^2^	0.002	0.009 **		(Maternal age)^2^	0.001	0.009 ****

* Time-continuous variable for time. ** Z1 = (Time−1918.33) whose slope gives the change of slope from Pre-pandemic to Pandemic period, where 1918.33 refers to 31 March 31 1918 $x_{+}=\max\left(x,0\right)$. *** Z2 = (Time−1921.164) whose slope gives the change of slope from pandemic to post-pandemic period where 1921.164 refers to 31 January 1921. **** Significant at 0.05 level of significance.
